# Development of Nanoparticles Incorporating a Novel Liposomal Membrane Destabilization Peptide for Efficient Release of Cargos into Cancer Cells

**DOI:** 10.1371/journal.pone.0111181

**Published:** 2014-10-24

**Authors:** Shoko Itakura, Susumu Hama, Takashi Ohgita, Kentaro Kogure

**Affiliations:** Department of Biophysical Chemistry, Kyoto Pharmaceutical University, Kyoto, Japan; Universidade Nova de Lisboa, Portugal

## Abstract

In anti-cancer therapy mediated by a nanoparticle-based drug delivery system (DDS), overall efficacy depends on the release efficiency of cargos from the nanoparticles in the cancer cells as well as the specificity of delivery to tumor tissue. However, conventional liposome-based DDS have no mechanism for specifically releasing the encapsulated cargos inside the cancer cells. To overcome this barrier, we developed nanoparticles containing a novel liposomal membrane destabilization peptide (LMDP) that can destabilize membranes by cleavage with intramembranous proteases on/in cancer cells. Calcein encapsulated in liposomes modified with LMDP (LMDP-lipo) was effectively released in the presence of a membrane fraction containing an LMDP-cleavable protease. The release was inhibited by a protease inhibitor, suggesting that LMDP-lipo could effectively release its cargo into cells in response to a cancer-specific protease. Moreover, when LMDP-lipo contained fusogenic lipids, the release of cargo was accelerated, suggesting that the fusion of LMDP-lipo with cellular membranes was the initial step in the intracellular delivery. Time-lapse microscopic observations showed that the release of cargo from LMDP-lipo occurred immediately after association of LMDP-lipo with target cells. Consequently, LMDP-lipo could be a useful nanoparticle capable of effective release of cargos specifically into targeted cancer cells.

## Introduction

In cancer therapy, a drug delivery system (DDS) that can specifically deliver various therapeutic agents to tumor tissue is a powerful tool for achieving cancer cell-specific therapy without adverse effects. Rapid progress in genomic and proteomic research has led to the identification of molecules mediating the development and progression of cancer. Thus, it has become possible to design more specific therapeutics against cancer cells. In general, highly specific drugs such as peptides, antibodies, proteins and nucleic acids are high molecular weight (HMW) agents. However, the clinical application of many HMW agents is limited due to their rapid degradation by enzymes in the blood as well as their inadequate delivery into cells at their targeting sites [1]. To resolve these problems, HMW agents must be encapsulated in nanoparticles such as liposomes or polymeric micelles capable of protecting the cargos from enzymatic degradation and achieving efficient intracellular delivery. In current nanoparticle-based DDS, modification with polyethylene glycol (PEGylation) is an essential strategy for their delivery to tumor tissue via enhanced permeability and retention (EPR) effects as PEGylated carriers can circulate for long periods [2], [3]. However, only ∼10% of the injected molecules reach the tumor tissue [4]. Therefore, there is room for improved delivery. Moreover, tumor delivery via EPR effects depends on the number of immature tumor vessels. Consequently, the delivery efficiency in cancers with low vascular density, such as pancreatic cancer, is less efficient [5].

Although the development of anticancer DDS has rapidly progressed, there is no alternative strategy capable of enhancing the delivery of nanoparticles by EPR effects. Therefore, it is necessary to maximize the therapeutic efficacy of the encapsulated anti-cancer agents to compensate for low delivery efficiency to tumor tissue. When anti-cancer agents are encapsulated into nanoparticles, therapeutic efficacy depends on the amount of free agents released from the nanoparticles. Hence, the release efficiency of cargos from nanoparticles is a key step in enhancing therapeutic effectiveness. To achieve cancer cell-specific toxicity without undesirable side effects, the cargos encapsulated into nanoparticles must be released only when the nanoparticles are delivered to tumor tissue, not during their circulation. In the past, to achieve tumor-specific release of cargos, various nanoparticles have been designed to make use of the tumor environment. For example, nanoparticles that can release in tumor-specific acidic extracellular environments have been developed [6]–[8]. However, when a nanoparticle responds to the extracellular environment, the cargo is released outside the cells. This design creates a new technical problem because many HMW agents such as nucleic acids and proteins must be delivered and released inside the tumor cells. In addition, specific release of a low molecular weight (LMW) anticancer agent inside the tumor cells showed more effective anticancer activity than extracellular release [9].

In this regard, a DDS carrier capable of releasing a cargo into cancer cells has been developed. For example, one nucleic acid carrier depends on protein kinase Cα (PKCα), which is overexpressed in cancer cells [10], [11]. This carrier is a complex of nucleic acids and cationic polymers incorporating a substrate sequence that can be targeted by PKCα. Their electrostatic interaction is reduced by the phosphorylation of the substrate sequence on the polymer by PKCα, after which the nucleic acid is released. A different approach takes advantage of the elevated ATP concentration in cancer cells. That is, an agent encapsulated in chaperonin is released following an ATP-mediated trigger [12]. However, in these release systems, the delivery of agents is limited and *in vivo* application is difficult. Thus, further improvements in pharmacokinetics and intracellular dynamics are required. In this regard, liposomes can encapsulate HMW drugs and LMW agents. Hydrophobic agents can be carried in the lipid membrane and hydrophilic agents can be retained in the aqueous phase [13]. Thus, the versatility of liposomes makes them intriguing for design of new DDSs.

Regarding the release of cargos from liposomes, many systems have been developed to induce the fusion of liposomal membranes with endosomal-lysosomal membranes in the acidic environment of the endosome-lysosome [14]. However, when the efficiency of escape from the endosome is low, drugs can undergo lysosomal degradation and endocytic recycling [15]. Therefore, improvements in drug release via membrane fusion are needed. Towards that end, we developed a system that destabilizes the liposomal membrane during fusion with the cancer cell membranes. We focused on the membrane intrinsic protein γ-secretase of cancer cells. This intramembranous protease can cleave the transmembrane domain of the substrate protein Notch [16], [17]. To use this mechanism, we incorporated peptides mimicking the transmembrane domain of Notch into the lipid membrane of the liposomes. Thus, when the liposome membrane partially fuses with the cell membrane, encapsulated cargos would be released into the cells by the destabilization of liposomal membranes by intramembranous γ-secretase. Using this concept, we incorporated a liposomal membrane destabilization peptide (LMDP) into liposomes (LMDP-lipo) that mimicked the transmembrane domain of Notch. Here, we demonstrate that release of cargos from LMDP-lipo was responsive to γ-secretase in cancer cell membranes.

## Materials and Methods

### Materials

Egg phosphatidylcholine was obtained from NOF Corporation (Tokyo, Japan). 1,2-dioleoyl-3-trimethylammonium propane (DOTAP) and 1,2-dioleoyl-sn-glycero-3-phosphoethanolamine (DOPE) were purchased from Avanti Polar Lipid (Alabaster, AL, U.S.A.). Cell Tracker CM-DiI was obtained from Invitrogen (Carlsbad,CA, U.S.A.). Cholesteryl hemisuccinate (CHEMS) and calcein were purchased from Sigma Aldrich (St. Louis, MO, U.S.A.). Bio-Beads SM-2 Adsorbents were purchased from BIO-RAD (Hercules, CA, U.S.A.). HUEhT-2 cells, an immortalized human umbilical vein endothelial cell (HUVEC) line established by electroporation of pIRES-hTERT-hygr, HeLa cells, a human cervical epithelioid carcinoma cell line, and MCF-7 cells, a human mammary tumor cell line, were obtained from the JCRB Cell Bank (Osaka, Japan). A549 cells, a human lung adenocarcinoma cell line, were provided by the Riken Cell Bank (Saitama, Japan). The liposomal membrane destabilization peptide (LMDP), LHLMYVAAAAFVLLFFVGCGVLLSRKRRR, was synthesized by SCRUM (Tokyo, Japan). A fluorescence-quenching peptide substrate, Nma-GGVVIATVK (Dnp) RRR-NH_2_ and an inhibitor of γ-secretase, N-[N-(3,5-Difluorophenacetyl)-L-alanyl]-S-phenylglycine t-butyl ester (DAPT), were obtained from the Peptide Institute, Inc. (Osaka, Japan). 3-[(3-Cholamidopropyl) dimethylammonio]-2-hydroxypropanesulfonate (CHAPSO) was purchased from Dojindo Laboratories (Kumamoto, Japan).

### Membrane isolation from cells

The membrane fraction was prepared according to previous reports [18]. All procedures were carried out on ice. Cultured HeLa cells, A549 cells, MCF-7 cells and HUEhT-2 cells (about 1×10^8^ cells) were pelleted. Cells were resuspended in a buffer containing 20 mM Hepes, pH 7.5, 50 mM KCl, 2 mM EGTA, and protease inhibitor cocktail, Complete Mini (Roche Applied Science), and homogenized using 15 strokes of a Dounce homogenizer with a tight pestle. The cellular homogenates were centrifuged at 800 × *g* for 10 min and rehomogenized as above. The supernatants were centrifuged at 100,000 × *g* for 1 h and resuspended in the same buffer and recollected by centrifugation at 100,000 × *g* for 30 min using an Optima XL-90 (BECKMAN COULTER). Membranes were resuspended in 20 mM HEPES, pH 7.0, 150 mM KCl, 2 mM EGTA, 1% (w/v) CHAPSO, and Complete Mini and solubilized at 4°C for 1 h with end-over-end rotation. The solubilized membranes were centrifuged at 100,000 × *g* for 1 h, and the supernatants were collected. The total protein concentration was determined with a BCA Protein Assay Kit (Thermo scientific).

### γ-secretase activity measurement

To measure γ-secretase activity, solubilized membrane preparations (15 µg total protein) were incubated with eight µM fluorescence-quenching peptide substrate at 37°C overnight and centrifuged at 16,100 × *g* for 15 min. The fluorescence of supernatants was measured by Plate Infinite M200 (TECAN) with an excitation wavelength of 355 nm and emission wavelength of 440 nm.

### Preparation of LMDP-lipo

Thin lipid films composed of EPC, EPC/DOPE/DOTAP (4/4/1), EPC/DOPE/CHEMS (4.5/4.5/2) in a glass tube were hydrated in PBS (−) or 30 mM calcein solution in PBS (−) for 10 min, followed by sonication for 5 min in a bath-type sonicator (NEY). The liposomes were mixed with 1% Triton X-100 and incubated end over end for 1 h. One mM LMDP dissolved in 1% Triton X-100 was added to the liposome solution (3–5 mol%) and incubated end over end for 1 h. Then, SM-2 Bio-beads were added to the lipid-LMDP-detergent solution for 1 h at a beads-to-detergent ratio of 30 (w/w) in order to remove detergent. The resultant solution was centrifuged at 16,000 × *g* for 10 min and the supernatant liposome solution was collected. For liposomes containing calcein, free calcein was removed using a Sephadex G-50 column equilibrated with PBS (−). The lipid concentration of liposomes was determined with the choline oxidase-DAOS method (phospholipids C-test Wako kit). Particle size and surface potential of prepared liposomes were measured with a Zetasizer nano (Malvern Ins. Ltd.).

### LMDP cleavage activity by γ-secretase

To study LMDP cleavage by γ-secretase, the competitive inhibitory effect of LMDP or LMDP-lipo on the cleavage of the peptide substrate (the same as that used in 2.2) was evaluated. The solubilized membrane preparations of A549 (16.5 µg total protein) were incubated with 5 µM peptide substrate and 0, 5, 10, 50 or 100 µM LMDP or LMDP-lipo as the LMDP concentration were held constant at 37°C overnight. Then, the fluorescence intensity was measured as described in 2.2.

The cleavage of LMDP was also evaluated by HPLC assay. The solubilized membrane preparations of A549 (16.5 µg total protein) were incubated with 100 µM LMDP or LMDP-lipo at 37°C for 1 h. LMDP at a concentration of 100 µM had inhibitory effects in competitive inhibition studies and the incubation time (1 h) was matched with the reaction condition in the calcein release assay. LMDP-lipo was solubilized with 1% Triton X-100, and the LMDP remaining in solution was detected by high-performance liquid chromatography (HPLC) using Alliance system (Waters, Milford, MA, USA) connected to a column of SunFire C18 (5 µm, 4.6 × 150 mm, Waters, Milford, MA, USA). The mobile phase consisted of 0.1% TFA in H_2_O (mobile phase A)/and acetonitrile in 0.1% TFA (mobile phase B). The gradient was started with an initial ratio of 80% mobile phase A and 20% mobile phase B, followed by a linear gradient from 80 to 10% mobile phase A for 20 min. The flow rate was 0.3 mL/min and the detection wavelength was 220 nm.

### Calcein release assay

To study the release of cargos from liposomes, Control-lipo or LMDP-lipo containing calcein were incubated with solubilized membranes (lipid/protein = 2.0 (w/w)) or PBS or 1% Triton X-100 at 37°C for 1 h in the presence or absence of the γ-secretase inhibitor DAPT. The solubilized membranes were preincubated with γ-secretase inhibitor DAPT (10 µM) at 37°C for 30 min before treatment with liposomes. The released calcein was assayed by measuring the intensity of fluorescence using a PLATE Infinite M200 (TECAN) with an excitation wavelength at 490 nm and emission wavelength at 515 nm. The percentage of calcein release was calculated by the following formula:

Calcein Release (%) = [(F−F_0_)/(F_100_−F_0_) ] × 100, where F, F_0_ and F_100_ were the fluorescence intensities of calcein after the incubation in the presence of a membrane fraction, in PBS and in 1% Triton, respectively.

### Confocal laser scanning microscopic observations of LMDP-lipo

A549 cells were cultured on 0.002% poly-L-lysine- (PLL) coated 96-well imaging plates (BD Falcon) at a density of 1×10^4^ cells/well for 24 h in DMEM containing 10% fetal bovine serum (FBS). After washing with PBS (−), cells were treated with Control-lipo or LMDP-lipo containing 0.2 mol% CM-DiI and 30 mM calcein for 1 h in serum-free DMEM. CM-DiI and calcein were excited with He/Ne (543 nm) and Ar (488 nm) lasers, respectively. A series of images was obtained by confocal laser scanning microscopy (CLSM) (LSM 510 META, Carl Zeiss Co. Ltd., Jena, Germany) equipped with an oil immersion objective lens (Plan-Apochromat 63/NA1.4). To inhibit γ-secretase activity and the endocytotic pathway, A549 cells were cultured as above and the cells were pre-incubated in serum-free DMEM containing 50 µM DAPT or 0.4 M sucrose at 37°C or 30 min, 2.5 mM amiloride at 37°C for 10 min and 5 µg/mL FilipinIII at 37°C for 1 h, followed by treatment with liposomes at 37°C for 1 h and observed by CLSM, as described above. For a detailed study of calcein release, liposomes were observed by time-lapse imaging of liposomes in living A549 cells. The cells were cultured on PLL-coated 35 mm glass-bottom dishes (IWAKI) at a density of 2×10^5^ cells/dish for 24 h in DMEM containing 10% FBS. The cells were washed with PBS (−) and treated with LMDP-lipo containing 0.2 mol% CM-DiI and 30 mM calcein at 4°C for 10 min in serum-free DMEM. After removal of liposomes, the cells were washed 3 times with PBS (−) followed by addition of fresh serum-free DMEM. Time lapse imaging was acquired using a Nikon A1 CLSM (Nikon Instruments Inc., Melville, USA) equipped with an oil-immersion objective lens (Plan Apo VC 60X 1.4 N.A.). The samples were maintained at 37°C and 5% CO_2_ during all imaging experiments. Laser light at 488 nm and 561 nm were used to excite calcein and DiI, respectively. Time-lapse acquisition was configured to take images of the same field every 30 sec over 30 min. The time lapse imaging of about 3 dots/cell for 5 cells of each sample was analyzed using NIS-Elements software (Nikon Instruments Inc., Melville, USA).

### Statistical analysis

Statistical significance was determined using Student’s t-tests and Tukey-Kramer HSD-tests. Differences with P-values of less than 0.05 were considered significant.

## Results

### System design: release of cargos into cells through γ-secretase-dependent cleavage of LMDP incorporated into the liposomal membrane

**Figure 1:**
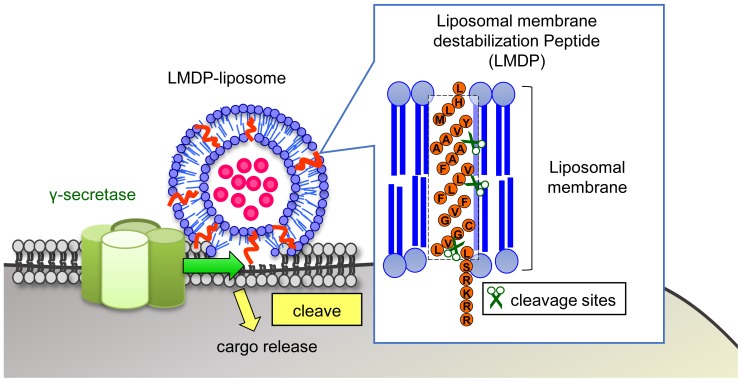
Development of nanoparticles incorporating a novel liposomal membrane destabilization peptide (LMDP) for efficient drug release into cancer cells. This image depicts the concept of the carriers incorporating LMDP into the liposomal membrane (LMDP-lipo). The lipid bilayer membrane of LMDP-lipo is destabilized by cleaving LMDP as a trigger for membrane fusion. The destabilization promotes cargos release into the cell.

We devised a system in which a substrate peptide of γ-secretase was incorporated into liposomal membranes. Such liposomes should be capable of carrying a cargo and efficiently releasing it into cancer cells. The peptide, as shown in Fig. 1, was composed of 29 amino acid residues that comprised a hydrophilic site inside the cell and a hydrophobic sequence of the transmembrane domain of Notch. It was termed “Liposomal Membrane Destabilization Peptide” (LMDP). The secondary structure of LMDP was predicted using SOSUI software [19]. As illustrated in Fig. 1, it was shown that 23 residues of the N-terminus assumed a helical structure and penetrated the membrane, and 6 residues of the C-terminal side were highly hydrophilic and were located outside of the lipid membrane. Therefore, the LMDP was designed to penetrate the lipid bilayer. In addition, the LMDP could be cleaved at 3 points by γ-secretase [17]. In order to determine the specificity of LMDP for γ-secretase, we performed an homology search of LMDP by BLAST. There was no protein having high homology with LMDP. Thus, LMDP might not be a substrate for proteases highly expressed by cancer cells such as matrix metalloproteinase. Thus, when incorporated into liposomes (LMDP-lipo), cleavage of LMDP should destabilize the lipid bilayer by triggering membrane fusion, followed by release of cargos into the cell.

### The cleavage of LMDP by γ-secretase

**Figure 2:**
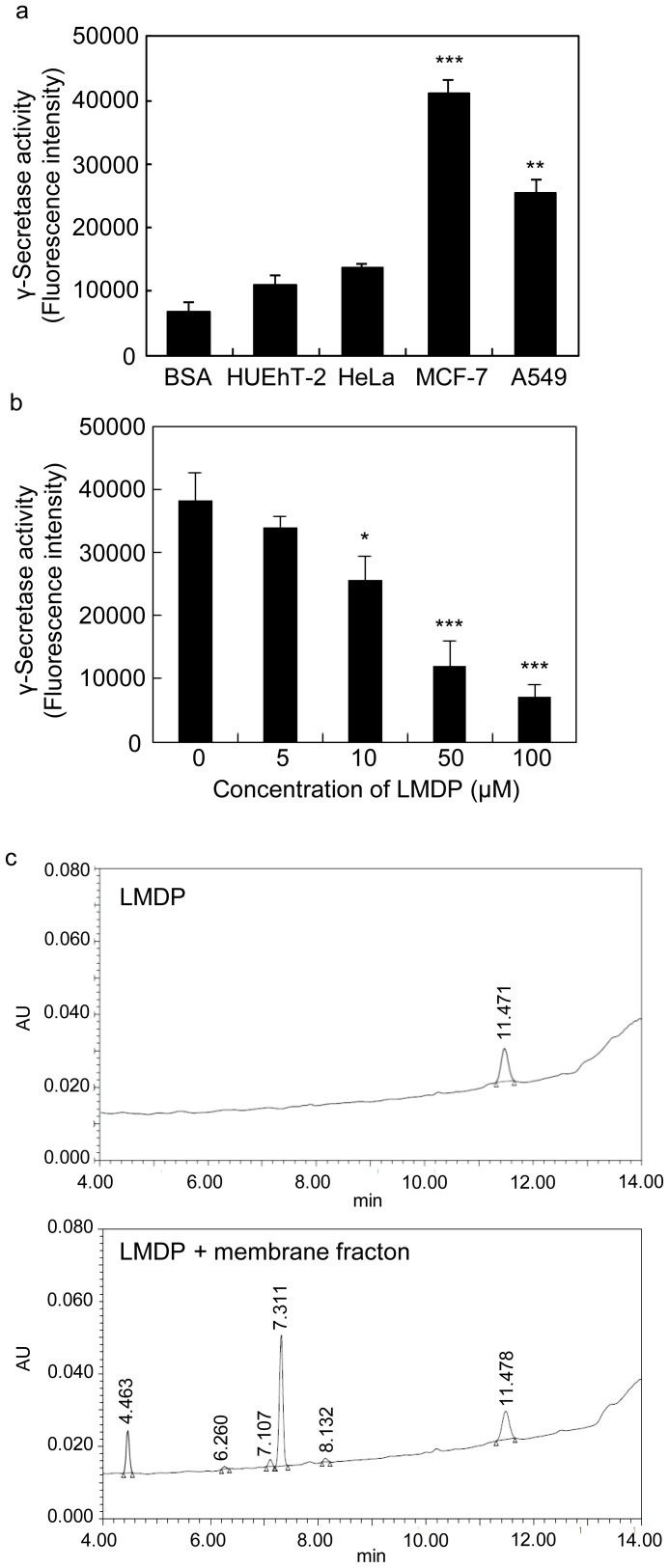
LMDP cleavage by γ-secretase. (a) γ-secretase activity in cultured cells. The fluorescence peptide probe was incubated with membrane fractions from HUEhT-2, HeLa, MCF-7 and A549 cells. (b) Competitive inhibition of LMDP. The peptide probe and LMDP were incubated at the indicated concentrations in the membrane fraction of A549 cells at 37°C overnight. Values and bars represent the means and SD, respectively. (c) Representative chromatograms of LMDP with or without the membrane fraction as determined by HPLC analysis from three independent experiments. *, P<0.05; **, P<0.01; ***, P<0.001 versus HUEhT-2 (a), 0 µM LMDP (b), n = 3.

To confirm that the LMDP was cleaved by γ-secretase, we chose A549 (human lung carcinoma), MCF-7 (human breast cancer) and HeLa (human cervical cancer) cell lines in which high activity of γ-secretase had been reported [20]–[22]. The γ-secretase activity in the membrane fraction of these cells was investigated using fluorescent substrate peptide probes that could be cleaved by γ-secretase. In addition, HUEhT-2 (a human vascular endothelial cell line) was also examined as a normal control. Before cleavage, fluorescence of the substrate peptides was quenched due to fluorescence resonance energy transfer (FRET) between a quenching group (Dnp) and a fluorescent group (Nma) at the N-terminus of the peptide across the cleavage site. Thus, fluorescence would be appeared by elimination of FRET due to cleavage of the peptides. Such peptide probes are often used for evaluation of γ-secretase activity [18]. We found that the γ-secretase activities were almost the same in HeLa cells and normal HUEhT-2 cells, but were significantly higher in MCF-7 and A549 cells (Fig. 2a). In addition, the expression of presenilin-1, which is an active center of γ-secretase, was examined by flow cytometry. As shown in Fig. S1, expression of presenilin-1 was observed at the same levels in all cells. The specific activity of γ-secretase (γ-secretase activity/presenilin-1 expression) was calculated and γ-secretase activity was higher in MCF-7 and A549 cells than in other cell lines. Since A549 cells had larger plasma membrane areas than MCF-7 cells, they were preferred for intracellular release assays.

The membrane fraction was isolated from A549 cells and was assessed for LMDP cleavage activity. To assess the interaction of LMDP with γ-secretase, we examined the competitive inhibition of LMDP on the cleavage of the fluorescent substrate peptide probe by a membrane fraction containing the γ-secretase. The fluorescence intensity of the substrate probe was significantly reduced in a LMDP concentration-dependent manner (IC_50_∶29 µM) (Fig. 2b). This result suggested that cleavage of fluorescent substrate peptide with γ-secretase was competitively prevented by LMDP. A significant decrease in fluorescence intensity was not observed when it was examined in the same way using a different polypeptide that had a similar size (30 residues) (Fig. S2). Those results suggested that the LMDP sequence was specifically recognized by the γ-secretase.

The cleavage of LMDP by γ-secretase was next examined by HPLC. The peak area of LMDP was reduced about 22.6±10.5% in the presence of a membrane fraction having γ-secretase activity (Table 1), thereby confirming the cleavage of LMDP. Fig. 2c shows a representative chromatogram of LMDP treated with or without the membrane fraction. These results suggested that synthetic LMDP was cleaved by an A549 cell membrane fraction possessing high γ-secretase activity.

**Table 1:** LMDP cleavage activity of γ-secretase assessed by HPLC analysis.

Sample	peak area (µV/sec)	cleavage efficiency (%)
LMDP	142557±23868	–
LMDP+membrane fraction	108204±32792	22.6±10.5

### Construction of LMDP-lipo

**Figure 3:**
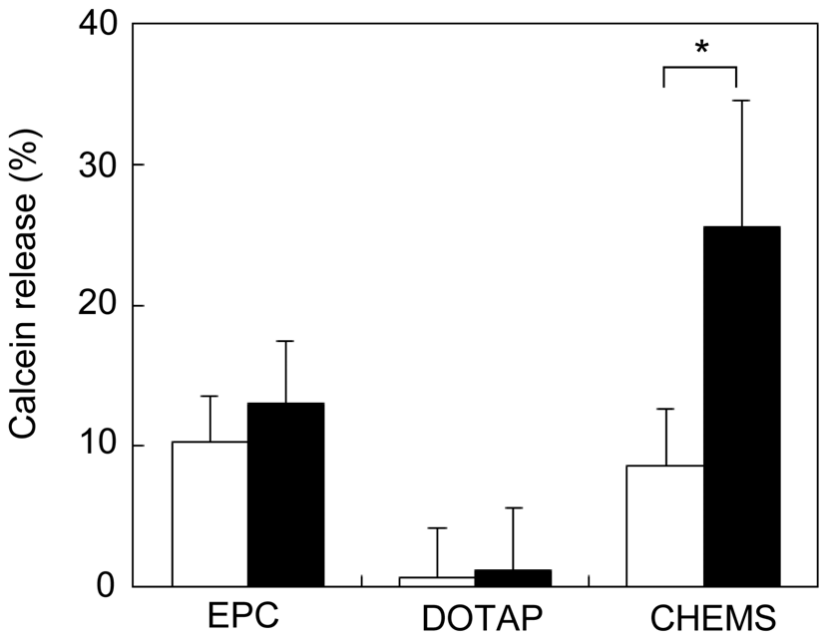
Calcein release assay. Control-lipo consisted of EPC or EPC/DOPE/DOTAP (DOTAP), or EPC/DOPE/CHEMS (CHEMS). LMDP-lipo contained 5 mol% LMDP. They were incubated with the membrane fraction from A549 cells at 37°C for 1 h. White and black columns indicate Control-lipo and LMDP-lipo, respectively. Values represent the means of three individual experiments. Bars represent SD. *, P<0.05.

LMDP was incorporated into liposomal membranes by a detergent removal method. LMDP was solubilized with a surfactant (1% Triton X-100) and was mixed with phospholipid. Then, the surfactant in the lipid/LMDP mixture was removed to prepare liposomes incorporating LMDP by a bead adsorption method as described in the Materials and Methods. We determined that 2.3±0.2% LMDP was incorporated in the liposomes by HPLC analysis. Calcein was used as a model cargo and was encapsulated within the liposomes. We assessed the leakage of calcein in the presence of an A549 cell membrane fraction. When incorporating LMDP into liposomes consisting of EPC, there was no difference in the leakage rate of calcein compared with liposomes without LMDP (Fig. 3). In this system, the transmembrane domain of the substrate peptide in the liposomal membranes has to be cleaved by γ-secretase in the cell membrane. Because it was necessary to optimize the lipid composition of the liposomes, we attempted to give the liposomes membrane fusion capability.

Thus, DOPE was included in the lipid composition. Moreover, since LMDP had a hydrophobic transmembrane domain and a hydrophilic site with basic amino acids (arginine and lysine) (Fig. 1), we conjectured that the electrostatic interaction between the surface charge of the liposomes and the charge of the hydrophilic site would affect both the incorporation of LMDP into liposomes and their functionality. Thus, we prepared membrane fusion liposomes with positive or negative charges containing DOTAP or CHEMS in EPC/DOPE and incorporated the LMDP into each.

The leakage of the encapsulated calcein from the negatively charged EPC/DOPE/CHEMS liposomes increased three-fold in the presence of a membrane fraction containing γ-secretase. On the other hand, when the composition included the cationic lipid DOTAP, leakage of calcein was not increased by LMDP (Fig. 3). The results suggested that inclusion of CHEMS and DOPE with EPC was required to optimize the functionality of the LMDP. The physicochemical properties of the liposomes and their compositions are summarized in Table 2.

**Table 2:** Lipid composition and physicochemical properties of LMDP-lipo.

Lipid composition		Size (nm)	Z-potential (mV)	PdI
EPC	Control	197±30	0.83±3.0	0.23±0.03
	LMDP	171±67	−1.1±2.8	0.26±0.13
EPC/DOPE/DOTAP	Control	148±52	12.6±4.3	0.27±0.09
	LMDP	112±19	7.5±0.7	0.25±0.16
EPC/DOPE/CHEMS	Control	126±12	−14.4±5.2	0.14±0.02
	LMDP	133±20	−9.0±3.8	0.21±0.07

### The mechanism of drug release by LMDP-lipo

**Figure 4:**
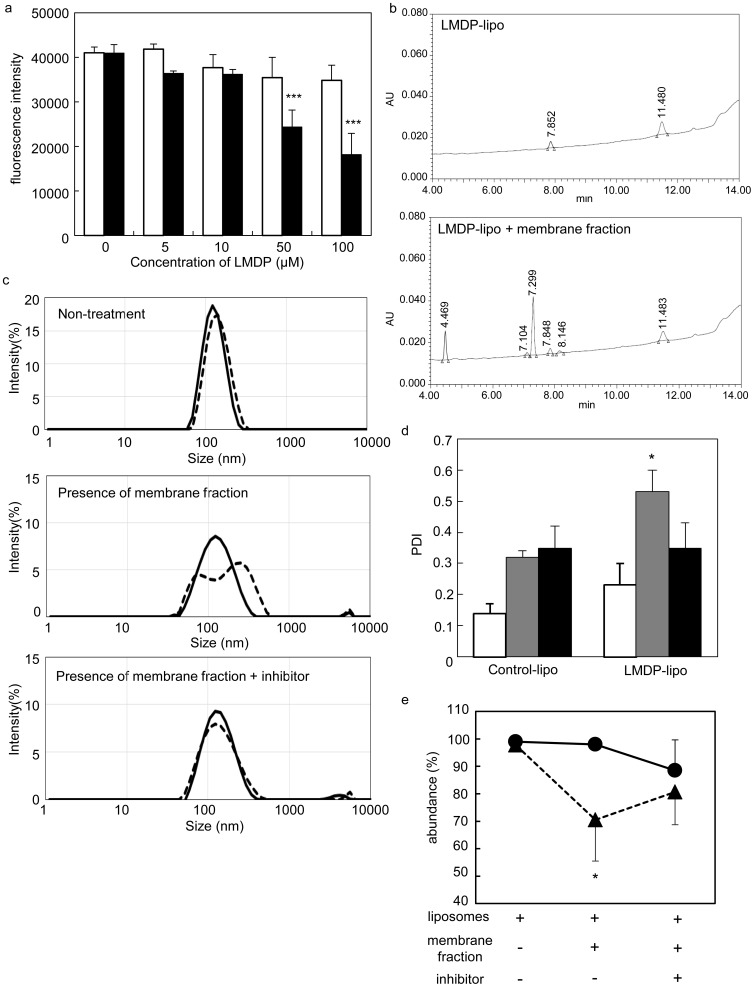
Characterization of LMDP-lipo cleavage by γ-secretase. (a) Competitive inhibition of LMDP in liposomes. The fluorescence peptide probe was incubated with Control-lipo (EPC/DOPE/CHEMS) or LMDP-lipo (EPC/DOPE/CHEMS/3 mol% LMDP) at the indicated LMDP concentrations in the presence of the membrane fraction of A549 cells at 37°C overnight. White and black columns indicate Control-lipo or LMDP-lipo, respectively. (b) Representative chromatograms of LMDP-liposomes with or without the membrane fraction as determined by HPLC analysis from three independent experiments. (c) Size distribution of liposomes in the presence of the membrane fraction obtained from A549 cells with or without γ-secretase inhibitor was measured using the Zetasizer nano. Solid line and dotted line indicate Control-lipo and LMDP-lipo, respectively. (d) Polydispersity index (PDI) of the peak using Control-lipo or LMDP-lipo. White, gray and black columns indicate liposomes alone, liposomes in the presence of an A549 cell membrane fraction or liposomes in the presence of a membrane fraction with 10 µM DAPT, respectively. (e) Abundance distribution of the peak for Control-lipo (black circles) and LMDP-lipo (black triangles) under the same conditions as (d). (Values and bars represent the means and SD, respectively. *, P<0.05 and ***, P<0.001 versus 0 µM LMDP (a), Control-lipo (c, d), n = 3.

We first determined whether LMDP was cleaved by γ-secretase when it was incorporated into liposomes. Thus, we tested the ability of LMDP-lipo to competitively inhibit the cleavage of a fluorescent substrate probe by γ-secretase. We verified that the cleavage of the fluorescent peptide probe was significantly inhibited even when LMDP-lipo was added to the membrane fraction containing the γ-secretase (Fig. 4a). Although the inhibitory effect of LMDP-lipo was less than LMDP alone, it was confirmed that LMDP in liposomal membranes could react with γ-secretase. The cleavage of LMDP in liposomal membranes was also evaluated by HPLC in the presence of the γ-secretase-containing membrane fraction. The peak area of LMDP in the liposomes was decreased by about 36.8±7.0%, and the cleavage of LMDP is shown in the presence of a membrane fraction (Table 3). Fig. 4b shows the representative chromatograms of LMDP-liposome with or without the membrane fraction.

**Table 3:** HPLC analysis of LMDP in liposomes after treatment with cell membranes containing γ-secretase.

Sample	peak area (µV/sec)	cleavage efficiency (%)
LMDP-liposome	110608±38188	–
LMDP-liposome+membrane fraction	67823±20184	36.8±7.0

The constitution of LMDP-liposome was EPC/DOPE/CHEMS (4.5∶4.5∶2) containing 3 mol% LMDP.

Next, we evaluated the change of LMDP-lipo particle size in the presence of the γ-secretase-containing membrane fraction. We also evaluated the influence of particle size in the presence of N-[N-(3, 5-difluorophenacetyl)-L-alanyl]-S-phenylglycine t-butyl ester (DAPT), a γ-secretase inhibitor. The inhibitory effect of DAPT was confirmed by using a substrate peptide probe. Specifically, γ-secretase activity was inhibited by about 60% by the addition of DAPT (Fig. S3). Fig. 4b shows that the particle size distribution of LMDP-lipo was broad compared to Control-lipo in the presence of a membrane fraction. This broad distribution was inhibited by treatment with DAPT. The polydispersity index (PDI) of LMDP-lipo was significantly increased in the presence of the membrane fraction compared to Control-lipo, a difference that was reduced by treatment with DAPT. Additionally, the PDI in Control-lipo treated with the membrane fraction was slightly increased in comparison with that in Control-lipo without the membrane fraction. However, this increase was not changed by co-treatment with the γ-secretase inhibitor, DAPT, suggesting that the increase of PDI in Control-lipo was due to the presence of the membrane fraction. (Fig. 4c). The abundance distribution of the major peak of the liposomes was compared in the absence or presence of the membrane fraction. The abundance of the major peak of LMDP-lipo was significantly decreased in the presence of the membrane fraction. These changes were suppressed by DAPT, and it was not observed in the Control-lipo (Fig. 4d). These results suggested that the size distribution was altered because LMDP was cleaved by γ-secretase, causing destabilization of the liposomal membrane and changing particle size.

### γ-secretase-dependent release of cargos from LMDP-lipo

**Figure 5:**
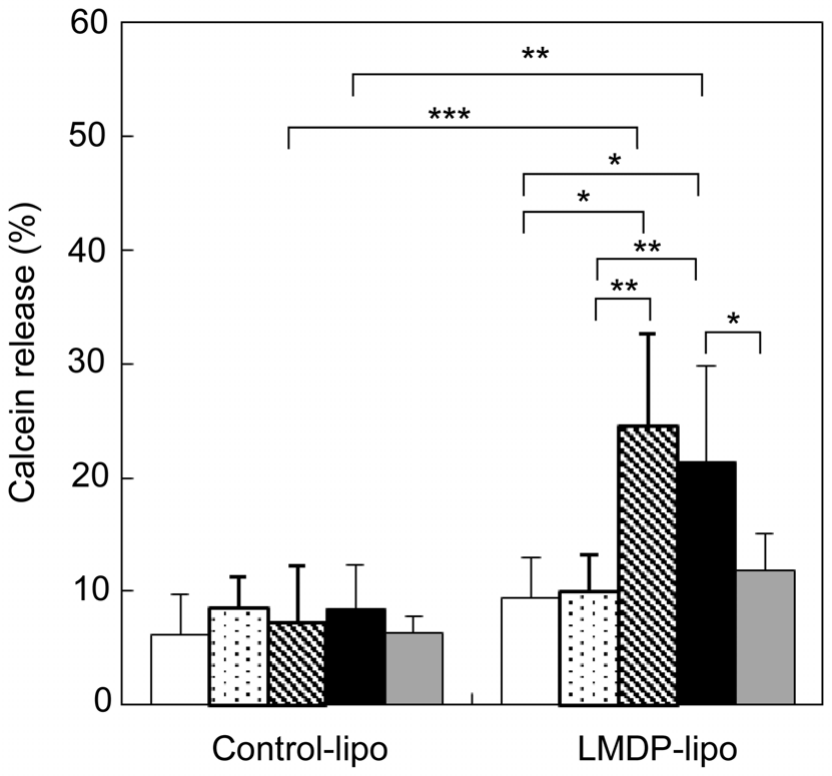
Selective calcein release from LMDP-lipo. Control-lipo (EPC/DOPE/CHEMS) or LMDP-lipo (EPC/DOPE/CHEMS/5mol% LMDP) was incubated in the presence of the membrane fraction from HUEhT-2 cells (white columns), HeLa cells (dotted columns), MCF-7 cells (cross-hatched columns), A549 cells (black columns), or A549 cells with 10 µM DAPT (gray columns) at 37°C for 1 h. Data represent means ± S.D. (n = 3–7) *, P<0.05, **, P<0.01 and ***, P<0.001.

We examined whether release of cargos depended on γ-secretase activity. Thus, LMDP-lipo encapsulating calcein was mixed with the membrane fraction from A549 cells, HeLa cells, MCF-7 cells or HUEhT-2 cells. When LMDP-lipo was mixed with membrane fractions isolated from A549 cells and MCF-7 cells with very high γ-secretase activities, the leakage of calcein increased compared to Control-lipo. The increased leakage was significantly suppressed by treatment with DAPT. On the other hand, calcein leakage did not increase when membrane fractions of HeLa cells and normal HUEhT-2 cells (slightly high or low γ-secretase activity, respectively) were used (Fig. 5). Therefore, the results suggest that the release of cargos from LMDP-lipo is dependent on high γ-secretase activity in cancer cells.

### Intracellular release of cargos from LMDP-lipo

**Figure 6:**
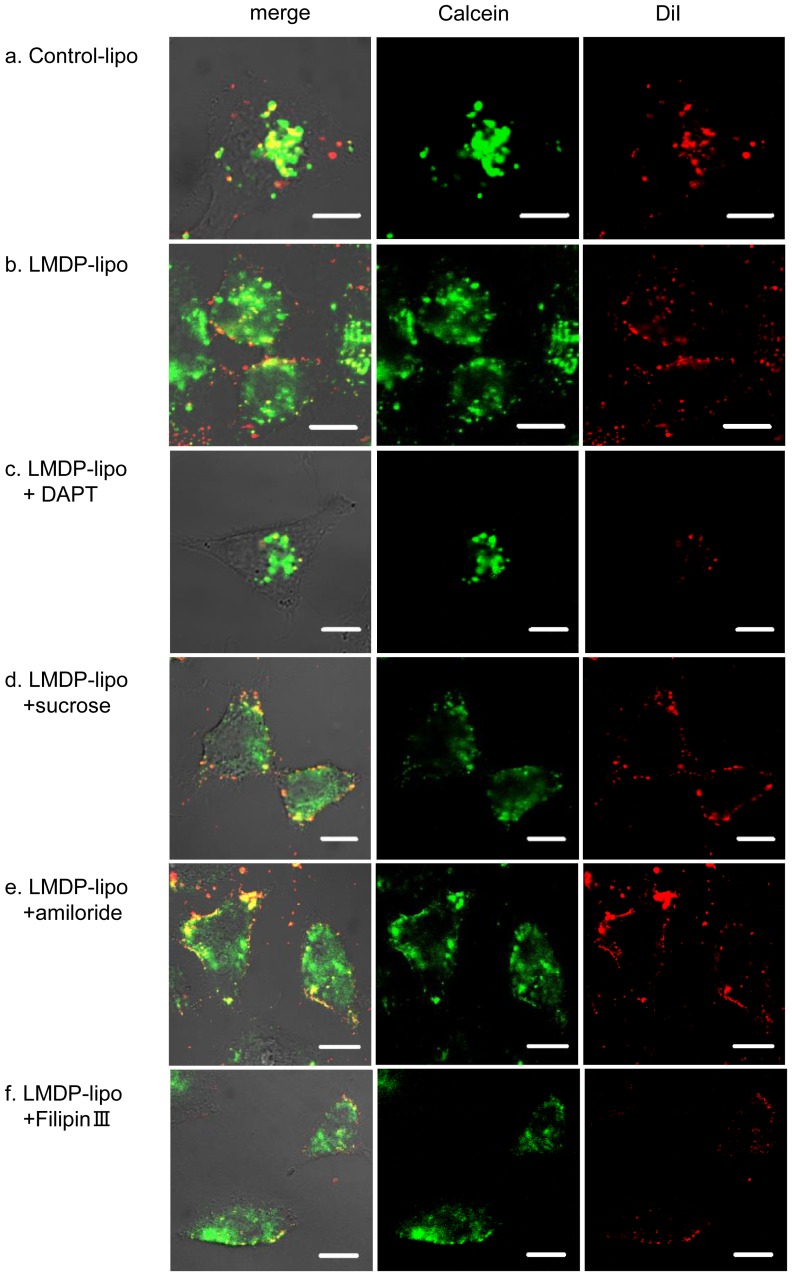
Calcein release from LMDP-lipo into cells. DiI-labeled and calcein encapsulated Control-lipo (EPC/DOPE/CHEMS) (a), LMDP-lipo (EPC/DOPE/CHEMS/5 mol% LMDP) (b), LMDP-lipo in the presence of 50 µM DAPT (c), LMDP-lipo in the presence of 0.4 M sucrose (d), LMDP-lipo in the presence of 2.5 mM amiloride (e) and LMDP-lipo in the presence of 5 µg/mL FilipinIII (f) were added to A549 cells and incubated at 37°C for 1 h. The intracellular location of CM-DiI (red) and calcein (green) was observed by CLSM. Red signal indicates liposomes. Scale bars, 10 µm.

We next examined the transfer of cargos from LMDP-lipo into cultured cells using the A549 line. Calcein (green) release from LMDP-lipo, of which membranes were labeled with CM-DiI (red), was observed by confocal laser scanning microscopy (CLSM). It seems that calcein release was promoted by the increase of the incubation time because fusion lipids were contained in the liposomes. Therefore, in this study, cells were treated for 1 h in order to determine the specific release by γ-secretase. The calcein released from LMDP-lipo diffused more widely into the cells than Control-lipo (Figs. 6a, b). Whereas yellow dots indicating co-localization of calcein and liposomes in the cells were more frequently observed in Control-lipo, the red dots of LMDP-lipo were more localized to the cell membrane and co-localization with green of calcein was decreased. (Figs. 6a, b). The diffusion was suppressed by the γ-secretase inhibitor DAPT (Fig. 6c). These results showed that LMDP-lipo was able to release encapsulated calcein in response to γ-secretase in cultured cells.

**Figure 7:**
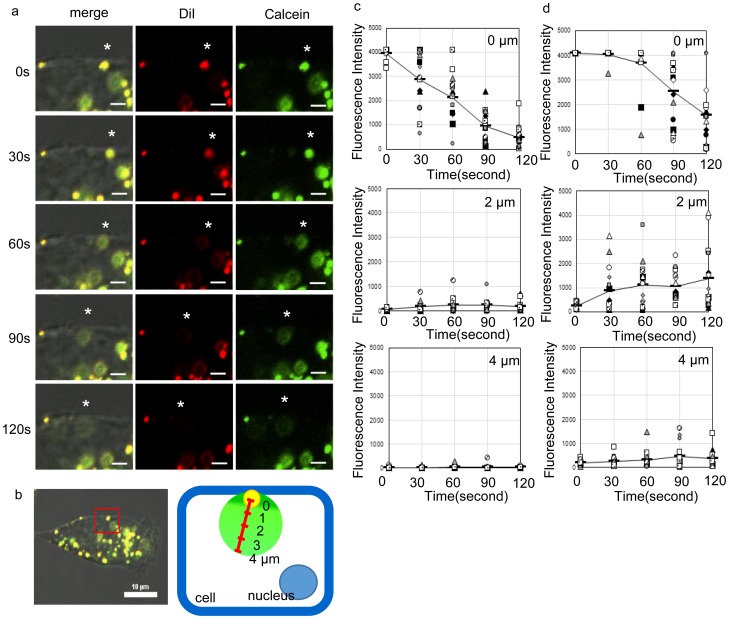
Time lapse imaging of LMDP-lipo by CLSM. (a) DiI-labeled and calcein-encapsulated LMDP-lipo (EPC/DOPE/CHEMS/5 mol% LMDP) was added to A549 cells and incubated at 37°C. CM-DiI (red) and calcein (green) were observed at intervals of 30 sec by CLSM. Asterisks indicate the same LMDP-lipo. Scale bars, 2 µm. (b) Schematic image of fluorescence measurement. Fluorescence intensities of CM-DiI and calcein were measured at 0, 2 and 4 µm from the centers of dots indicating fluorescent-labeled liposomes taken up by the cells. Changes in fluorescence intensity in 3 dots/cell for 5 cells at the indicated times were analyzed for CM-DiI (c) and calcein (d) using NIS-Elements software.

γ-Secretase is present not only in the plasma membrane but also in endosomal membranes [23]. Since DOPE and CHEMS have membrane fusion activity in the low pH endosomal environment [24]–[26], it is likely that the cargo is released into the cell by membrane fusion with endosomal membrane containing γ-secretase. In order to study release of cargos in the plasma membrane in detail, we evaluated the response when endocytic pathways were inhibited by sucrose, amiloride or FilipinIII as inhibitors of clathrin-mediated endocytosis, micropinocytosis or caveolae-mediated endocytosis, respectively. We found that the release of calcein from LMDP-lipo into the cells occurred in the presence of three major endocytosis inhibitors (Figs. 6d–f). Thus, the results confirmed that calcein was released from LMDP-lipo due to cleavage of LMDP by γ-secretase in the plasma membrane. To examine the kinetics of calcein release, we used time-lapse CLSM. LMDP-lipo labeled with CM-DiI and calcein as described above were added to A549 cells. Images were obtained every 30 sec to observe the release of calcein from liposomes over time (Fig. 7a and movie S1). We also evaluated the relationship between time and release (Fig. 7b) by determining the fluorescence intensity at fixed distances (0, 2, or 4 µm) from the center of the dot of liposomes on the plasma membrane. The fluorescence intensity of DiI as an indicator of the liposomal membrane was strong 0 µm from the center at 0 sec. It weakened gradually with time, and was reduced about 88% after 120 sec. Fluorescence was hardly observed 2 or 4 µm away from the center (Fig. 7c). We surmise that the fluorescent liposome lipids were diluted by fusion with the plasma membrane. In contrast, in the case of encapsulated calcein, fluorescence intensity was strong at 0 µm from the center at 0 sec and decreased with the passage of time such that fluorescence intensity decreased 68% after 120 sec. Moreover, the fluorescence intensity at 2 and 4 µm from the center increased with time (Fig. 7d). Thus, the calcein was initially co-localized with the liposomes but it was released due to fusion of the liposomes with the plasma membrane. The release of calcein from the liposomes was observed starting about 30 sec after contact with the cells (Fig. 7d), suggesting that LMDP-lipo released the cargos rapidly. These findings indicated that LMDP-lipo released encapsulated cargos into the cytoplasm of cells with high γ-secretase activity.

## Discussion

In this study, we developed liposomes that incorporated a substrate peptide (LMDP) for the purpose of destabilizing liposomal membranes. The functionality of this system was also demonstrated. Drug carriers using highly active secreted enzyme have been reported. For example, substrate degradation by elastase or matrix metalloprotease (MMP) can induce disruption or fusion of liposomal membranes [27], [28]. However, because these carriers release cargos outside of the cells, the low efficiency and the limitation on usable agents are concerns. Therefore, to achieve specific and efficient release of cargos inside of cancer cells, the carrier must be taken up into the cells with the encapsulated cargos yet avoid endosome recycling and lysosomal decomposition. To develop a system capable of cargos release when the carrier is taken up into cells, we focused on γ-secretase present in cancer cell membranes. γ-Secretase is a protease that plays an important role in Notch signaling. Notch controls the growth and differentiation of cells, and it is aberrantly activated in cancer cells [29], [30]. Notch signaling occurs when the transmembrane domain is cut by γ-secretase in the cell membrane and the intracellular fragment translocates to the nucleus [31], [32]. Therefore, γ-secretase is a protease essential for Notch signaling and its activity is enhanced in cancer cells [33], [34]. Evaluation of γ-secretase activity in several cell lines showed that activity was elevated two- to four-fold in cancer cells compared with normal HUEhT-2 cells (Fig. 2b). Therefore, we believe that LMDP-lipo could specifically release cargos inside cancer cells. The results in Fig. 3 showed that leakage of calcein increased in liposomes composed of EPC/DOPE/CHEMS compared to EPC alone or EPC/DOPE/DOTAP. It is suggested that γ-secretase in the cell membrane might not be able to access LMDP within the liposomal membrane consisting of EPC because EPC-based liposomes can form a stable lamellar structure. Thus, the membrane fusion capability of liposomes containing DOPE should likely enhance the accessibility of LMDP for γ-secretase in the cell membranes. The rate of calcein leakage from LMDP-lipo normalized to Control-lipo (leakage of LMDP-lipo/leakage of Control-lipo) was more than two-fold higher in A549 cancer cells than normal HUEhT-2 cells (Fig. 5). These results suggest that the liposomes could selectively release the cargos into cancer cells with high γ-secretase activity.

The enhanced γ-secretase-mediated release of calcein from LMDP-lipo was observed in cultured cells (Figs. 6 a–c). However, because we used DOPE/CHEMS that had membrane fusion activity at low pH, there was a concern that LMDP-lipo might release calcein in the endosomes. However, LMDP-lipo released calcein into the cells even in the presence of sucrose or amiloride or FilipinIII, which are inhibitors of clathrin-mediated endocytosis, micropinocytosis and caveolae-mediated endocytosis, respectively. (Figs. 6d–f). Those data suggest that incorporation of LMDP induced the release by contact with the plasma membrane and was not dependent on the low pH of the endosome. The release of calcein from LMDP-lipo was evaluated in detail by time-lapse analysis. It was observed that calcein diffused in about 2 min, as dots of liposomes contacted the cell membrane (Fig. 7). In general, the cytoplasmic delivery of nanoparticles through the endocytotic pathway is limited because nanoparticles are degraded in lysosomes and returned to the cell membrane by recycling. Polystyrene nanoparticles are transported from early endosomes to late endosomes in about 30 min following uptake by the cells, after which they are transported to lysosomes over the next 4 h [35]. The localization of lipid nanoparticles in the recycling endosome increases about 1 h after uptake, and then they are excreted out of the cell after about 6 h [36]. Our results suggest that LMDP-lipo released the encapsulated cargos rapidly into the cells via γ-secretase-mediated liposomal membrane destabilization when they fused with the plasma membrane or early endosomal membrane. Therefore, we suggest that this carrier has the capability to efficiently deliver cargos to the cytoplasm.

It will be necessary to modify LMDP-lipo with PEG for its *in vivo* application. However, the release efficiency of PEGylated LMDP-lipo may be lower than that of the unsubstituted LMDP-lipo because γ-secretase may have reduced access to the LMDP within the PEGylated lipo. In this regard, Hatakeyama and co-workers developed a PEGylated carrier capable of cleaving PEG from its surface in response to matrix metalloproteinase (MMP) overexpressed in cancer cells [37]. This system could improve the reduced affinity of the plasma membrane for a PEGylated carrier by the removal of PEG from the carrier surface outside of cells in response to MMP. By relying on MMP-induced cleavage of PEG, an efficient *in vivo* carrier should be possible.

## Conclusions

In this study, we developed a novel nanoparticle (LMDP-lipo) that used LMDP to destabilize the plasma membrane for release of cargos specifically within cancer cells. We found that release of cargos from LMDP-lipo into cancer cells depended on γ-secretase. Therefore, LMDP-lipo should be capable of effective release of cargos specifically inside cancer cells.

## Supporting Information

Figure S1 **Protein level of presenilin-1 in various cell lines.** Protein level of presenilin-1 was evaluated by flow cytometry. Cells (HUEhT-2, HeLa, MCF-7 and A549) were fixed and permeabilized. They were then treated with anti-presenilin-1 antibody or mouse IgG1 (Isotype control antibody) (one µg/1×10^6^ cells) followed by the secondary antibody (anti-mouse IgG Alxa488 F (ab′)_ 2_ fragment). Data are shown as mean fluorescence intensity ratio. Values represent the means of three individual experiments. Bars represent SD.
(TIF)

Figure S2 **Competitive inhibition by a peptide differing in sequence from LMDP.** The fluorescence peptide probe was incubated with a peptide (WEAALAEALAEALAEHLAEA LAEALEALAA) at the indicated concentrations in the presence of an A549 cell membrane fraction at 37°C overnight. Values represent the means of three individual experiments. Bars represent SD.
(TIF)

Figure S3 **Effect of γ-secretase inhibitor.** Membrane fraction of A549 cells was preincubated with DAPT (γ-secretase inhibitor) at the indicated concentrations at 37°C for 30 min and then incubated with the fluorescence peptide probe at 37°C for overnight. Values represent the means of three individual experiments. Bars represent SD. *P<0.05 and **P<0.01 versus 0 µM DAPT.
(TIF)

Movie S1 **Time lapse movie of the cells treated with LMDP-lipo.** A549 cells were treated with LMDP-lipo containing 0.2 mol% CM-DiI and 30 mM calcein at 4°C for 10 min in serum-free DMEM. After removal of liposomes, Time lapse imaging was acquired using a Nikon A1 CLSM (Nikon Instruments Inc., Melville, USA) equipped with an oil-immersion objective lens (Plan Apo VC 60X 1.4 N.A.). Laser light at 488 nm and 561 nm were used to excite calcein and DiI, respectively. Time-lapse acquisition was configured to take images of the same field every 30 sec over 10 min.
(AVI)
