# Supplementary figures and images for: Development of Nanoparticles Incorporating a Novel Liposomal Membrane Destabilization Peptide for Efficient Release of Cargos into Cancer Cells
Source: PLoS One. 2014 Oct 24;9(10):e111181. doi: 10.1371/journal.pone.0111181 (PMC4208851; doi:10.1371/journal.pone.0111181)

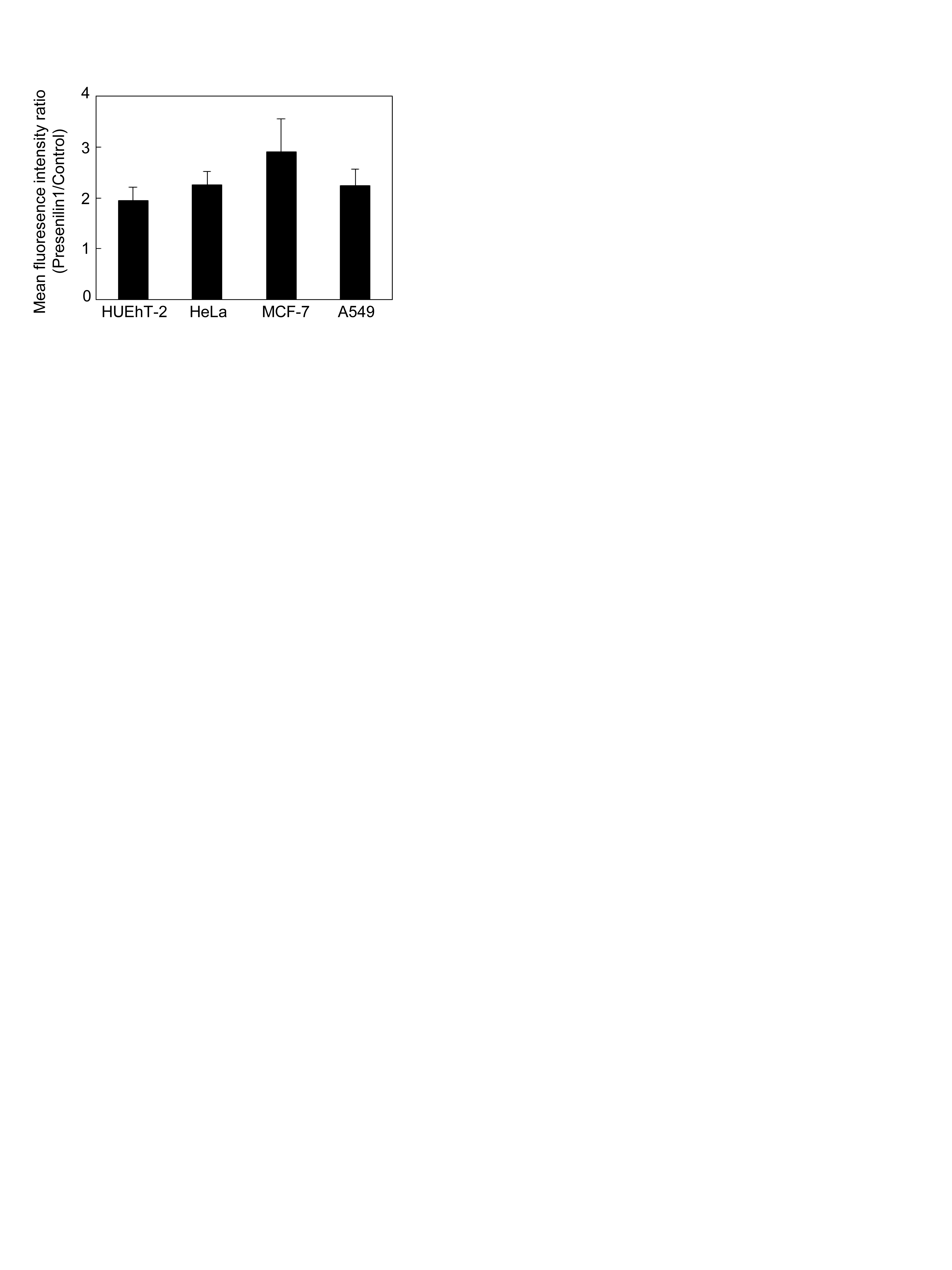

Supplement: Figure S1 — Protein level of presenilin-1 in various cell lines. Protein level of presenilin-1 was evaluated by flow cytometry. Cells (HUEhT-2, HeLa, MCF-7 and A549) were fixed and permeabilized. They were then treated with anti-presenilin-1 antibody or mouse IgG1 (Isotype control antibody) (one µg/1×106 cells) followed by the secondary antibody (anti-mouse IgG Alxa488 F (ab′) 2 fragment). Data are shown as mean fluorescence intensity ratio. Values represent the means of three individual experiments. Bars represent SD. (TIF) [file pone.0111181.s001.tif]

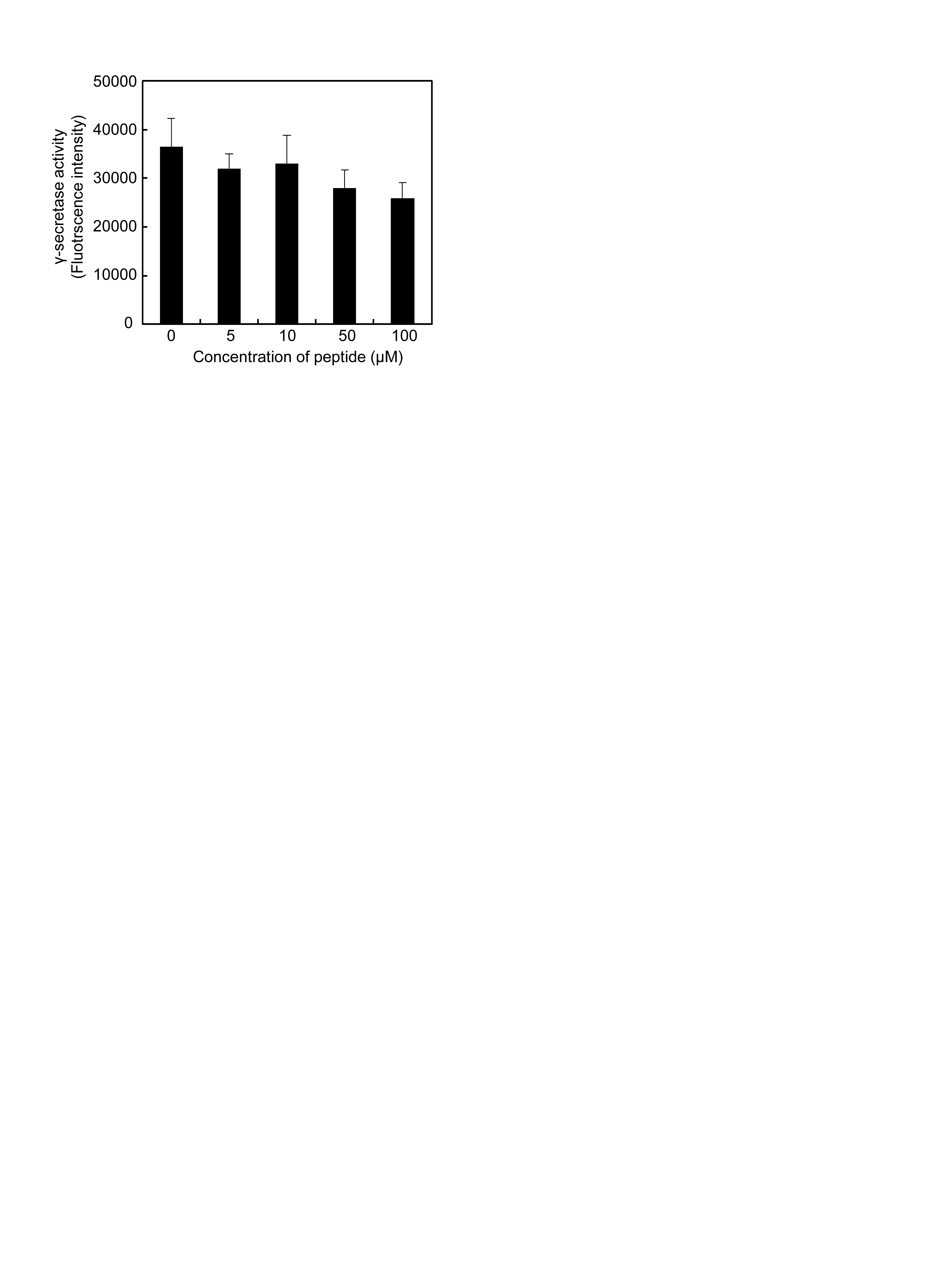

Supplement: Figure S2 — Competitive inhibition by a peptide differing in sequence from LMDP. The fluorescence peptide probe was incubated with a peptide (WEAALAEALAEALAEHLAEA LAEALEALAA) at the indicated concentrations in the presence of an A549 cell membrane fraction at 37°C overnight. Values represent the means of three individual experiments. Bars represent SD. (TIF) [file pone.0111181.s002.tif]

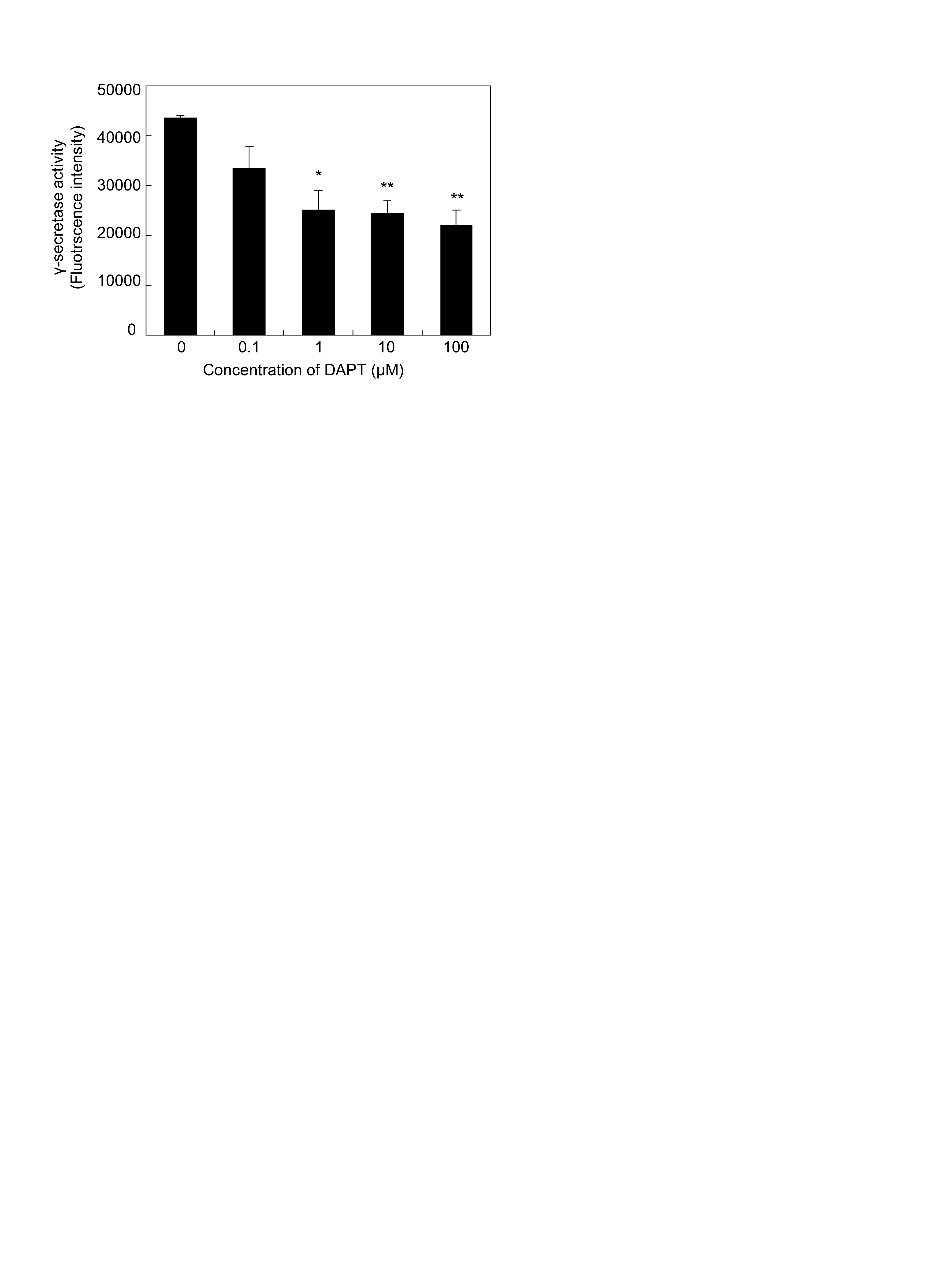

Supplement: Figure S3 — Effect of γ-secretase inhibitor. Membrane fraction of A549 cells was preincubated with DAPT (γ-secretase inhibitor) at the indicated concentrations at 37°C for 30 min and then incubated with the fluorescence peptide probe at 37°C for overnight. Values represent the means of three individual experiments. Bars represent SD. *P<0.05 and **P<0.01 versus 0 µM DAPT. (TIF) [file pone.0111181.s003.tif]
